# Correlated crustal and mantle melting documents proto-Tibetan Plateau growth

**DOI:** 10.1093/nsr/nwae257

**Published:** 2024-07-26

**Authors:** Wei Li, Rizheng He, Xiaohui Yuan, Felix Schneider, Frederik Tilmann, Zhen Guo, Yongshun John Chen

**Affiliations:** School of Geophysics and Geomatics, China University of Geosciences, Wuhan 430074, China; Deutsches GeoForschungsZentrum GFZ, Potsdam 14473, Germany; SinoProbe Laboratory, Chinese Academy of Geological Sciences, Beijing 100094, China; Deutsches GeoForschungsZentrum GFZ, Potsdam 14473, Germany; Deutsches GeoForschungsZentrum GFZ, Potsdam 14473, Germany; Deutsches GeoForschungsZentrum GFZ, Potsdam 14473, Germany; Freie Universität Berlin, Berlin 12249, Germany; Department of Ocean Science and Engineering, Southern University of Science and Technology, Shenzhen 518055, China; Department of Ocean Science and Engineering, Southern University of Science and Technology, Shenzhen 518055, China

**Keywords:** S-wave velocity, ambient noise tomography, partial melting, Tibetan Plateau, Hoh-Xil Basin

## Abstract

The mechanism that causes the rapid uplift and active magmatism of the Hoh-Xil Basin in the northern Tibetan Plateau and hence the outward growth of the proto-plateau is highly debated, more specifically, over the relationship between deep dynamics and surface uplift. Until recently the Hoh-Xil Basin remained uncovered by seismic networks due to inaccessibility. Here, based on linear seismic arrays across the Hoh-Xil Basin, we present a three-dimensional S-wave velocity (V_S_) model of the crust and uppermost mantle structure beneath the Tibetan Plateau from ambient noise tomography. This model exhibits a widespread partially molten crust in the northern Tibetan Plateau but only isolated pockets in the south manifested as low-V_S_ anomalies in the middle crust. The spatial correlation of the widespread low-V_S_ anomalies with strong uppermost mantle low-V_S_ anomalies and young exposed magmatic rocks in the Hoh-Xil Basin suggests that the plateau grew through lithospheric mantle removal and its driven magmatism.

## Teaser

Correlated partial melting imaged in the crust and uppermost mantle of the Hoh-Xil Basin suggests that lithospheric mantle removal and its driven magmatism promote the proto-Tibetan Plateau growth.

## INTRODUCTION

The Tibetan Plateau, as Earth's highest and largest orogenic plateau, has risen to an average altitude of ∼5 km with low internal relief (<1–2 km) and extended to a width of ∼2000 km after the collision between Indian and Asian continents started at ∼55 Ma [1,2]. Although many geophysical observations constrained the structure of the anomalously thick crust (∼60–80 km) of the plateau [3–5], little consensus has been reached on how such a plateau was formed and grew farther north from the India-Asia collision boundary.

According to surface tectonic observations, two primary end-member models have been proposed to explain the growth of the Tibetan Plateau, i.e. the stepwise rise of a sequence of terranes in the plateau [6,7] and the continuum deformation of the lithosphere in the entire plateau [8]. Geophysical observations have revealed deep dynamics in the orogenesis that have had a pronounced effect on forming the plateau, and two widely discussed hypotheses for plateau uplift are crustal channel flow [9,10] and lithospheric mantle removal [11]. The former argues that the plateau grew incrementally with the ductile flow of partially molten middle-lower crust [9,10], where the presence of melt was inferred from geophysically imaged low-velocity and high-conductivity zones [12,13] within the Tibetan middle-lower crust and Pliocene–Quaternary crust-derived felsic lavas in North Tibet [14,15]. For the lithospheric mantle removal model, the uplift of the northern Tibetan Plateau results from the delamination of an overthickened lithospheric mantle and asthenosphere upwelling [11], which is supported by the spatial coincidence of recent potassic volcanism [16,17] and an extensively imaged low-velocity and high-attenuation zone in the uppermost mantle [18–20]. Apparently, there is still much controversy concerning the growth mechanism of the Tibetan Plateau, in part due to the remaining unclear relationship between crustal structures and mantle dynamics beneath the plateau.

**Figure 1.**
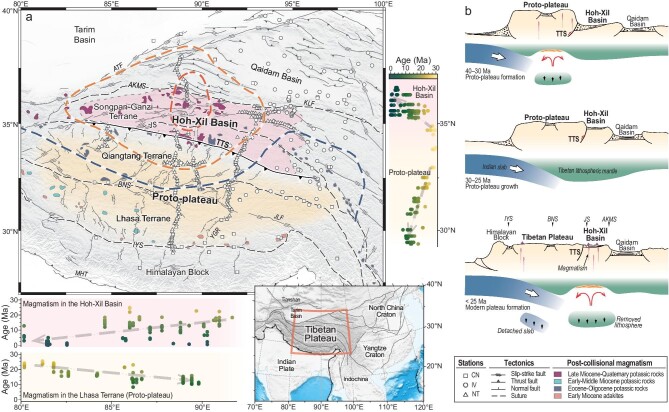
Tectonic setting of the Tibetan Plateau and stages of plateau growth. (a) Tibetan Plateau can be divided into Himalayan Block, Lhasa Terrane, Qiangtang Terrane, Songpan-Ganzi Terrane, and Qaidam Basin from south to north, which are separated by the Indus-Yarlung Suture (IYS), Bangong-Nujiang Suture (BNS), Jinsha Suture (JS), and Anymaqen-Kunlun-Muztagh Suture (AKMS). The pink-shaded area shows the Hoh-Xil Basin, which was once a foreland basin separated from the proto-plateau (orange-shaded area) by the Tanggula Thrust System (TTS). Major active faults are labeled, including Altyn Tagh Fault (ATF), Jiali Fault (JLF), Kunlun Fault (KLF), Main Himalayan Thrust (MHT), and Yadong-Gulu Rift (YGR). Colored patches mark post-collisional magmatic rocks sparsely exposed in the Tibetan Plateau with different episodes digitized from Chung *et al*. [16]. The ages of samples from these post-collisional magmatic rocks from Guo and Wilson [17] are projected onto longitudinal and latitudinal profiles, suggesting a young age from the central Tibetan Plateau to the south and north, but different E–W age propagations in the Hoh-Xil Basin and Lhasa Terrane, respectively. Thick dashed lines delineate the extreme uppermost mantle low-velocity zone (dark red) imaged in this study and the thin lithosphere (orange) and Indian slab's front (dark blue) interpreted by Xia *et al*. [34]. Squares, circles, and triangles denote seismic stations from the CDSN (CN), INDEPTH-IV (IV), and temporary seismic networks in North Tibet (NT) operated by the Chinese Academy of Geological Sciences. Inset shows the location of the study area. (b) The schematic model of the non-uniform growth across the Tibetan Plateau modified from Wang *et al*. [21] and Ding *et al*. [22]; 40–30 Ma, lithospheric delamination beneath South Tibet contributing to the formation of the proto-plateau; 30–25 Ma, Indian slab continued underthrusting and outward growth of the proto-plateau; <25 Ma, lithospheric delamination beneath North Tibet resulting in magmatism and rapid surface uplift of the Hoh-Xil Basin.

The non-uniform latitudinal growth of the Tibetan Plateau has been generally recognized by the temporal-spatial variations of surface uplift in the plateau from massive palaeo-altimetry data [21,22]. Tectonically the Tibetan Plateau is a collection of several terranes, i.e. Qaidam Basin, Songpan-Ganzi, Qiangtang, Lhasa, Himalayan Block, from north to south, which were gradually accreted to the south Asian continental margin since the Mesozoic [1] (Fig. 1a). Most of the Qiangtang Terrane and the Lhasa Terrane attained high elevations (>4 km) during the Eocene (∼55–45 Ma) and formed a proto-plateau with an intervening valley, which was uplifted in the Late Eocene–Oligocene (∼38–29 Ma) [22,23]. North of the proto-plateau along the Tanggula Thrust System (TTS), the Hoh-Xil Basin served as a foreland basin at relatively lower elevations (∼2 km) until it was rapidly uplifted in the Early Miocene (∼20 Ma) [21,24] (Fig. 1b). Cenozoic magmatism in the Tibetan Plateau also shows systematic temporal-spatial variations, indicating the diachronous growth of the plateau [16,17]. In particular, widespread felsic and potassic magmatism in the Hoh-Xil Basin since the Miocene [14–17] is coeval with the surface uplift as indicated by palaeo-altimetry data [21,23]. These young magmatic rocks and their entrained deep-derived xenoliths further indicate high temperature and partial melting in the crust and uppermost mantle [14,15,25,26]. It is therefore of great interest to explore the structure and thermal state of the crust and upper mantle beneath the Hoh-Xil Basin that is crucial to infer how the proto-plateau has grown northward since the Miocene.

Abundant seismic wave velocity models [13,19,20] for the northern Tibetan Plateau consistently show low-velocity zones (LVZs) in the crust and/or upper mantle, which contribute to the orogenesis models as mentioned above. However, the key area of the western Hoh-Xil Basin, which stands out due to the concentration of young magmatism coeval with rapid uplift in the Early Miocene (∼20 Ma), has been poorly covered by seismic stations so far [5]. Therefore, previous tomographic images did not provide good seismic constraints for the distribution and connection of LVZs in the crust and upper mantle, which can shed light on the possible mechanism responsible for plateau uplift and the young magmatism since the Miocene. Here, we make use of the recently available data recorded by seismic arrays located in the Hoh-Xil Basin (Fig. 1a). Our three-dimensional S-wave velocity (3-D V_S_) model is derived from ambient noise tomography. Ambient noise interferometry applied to a regional array recovers interstation surface wave dispersion information at short-to-intermediate periods [27] (6–65 s for this study), enabling resolution of the crust and uppermost mantle beneath the Tibetan Plateau. Particularly, new insights on the relationship between the partially molten crust and mantle dynamics beneath the Hoh-Xil Basin, when integrating with young magmatism there, could further illustrate the essential mechanisms for the plateau growth in the continental collision orogen.

## RESULTS

**Figure 2.**
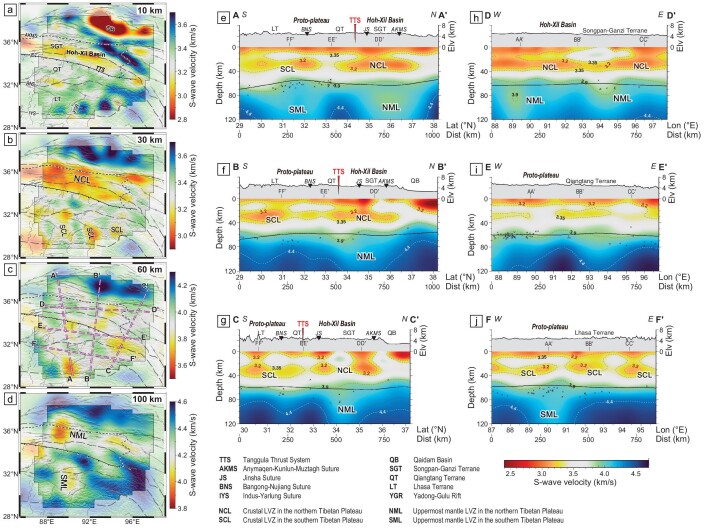
The 3-D V_S_ model constructed in this study. (a–d) Horizontal slices of the V_S_ model at depths of 10, 30, 60, and 100 km, respectively. (e–j) Vertical cross-sections of the 3-D V_S_ model along lines AA’, BB’, CC’, DD’, EE’, and FF’ indicated by the purple dashed lines in (c). Topography is plotted above each cross-section with labels representing the locations of sutures and terranes. Black crosses denote the Moho depths from the H-κ stacking of receiver functions [4,50] for stations located within a 50-km width corridor centered by the cross-section are plotted. Black lines indicate the Moho depths obtained by gravity analysis [30]. LVZs imaged in the crust are named as NCL and SCL, and those in the uppermost mantle as NML and SML, according to locations in the northern or the southern Tibetan Plateau.

**Figure 3.**
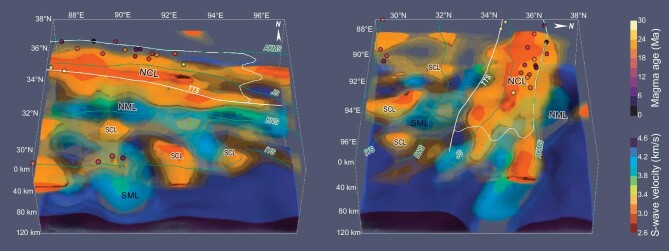
3-D visualizations of the V_S_ model. Iso-surfaces <3.35 km/s below 20-km depth and iso-surfaces <4.2 km/s below 80-km depth show LVZs in the crustal and uppermost mantle, respectively. Points mark the ages of exposed post-collisional magmatic rocks from Guo and Wilson [17]. Iso-surfaces in 3.20–3.35 km/s are translucently plotted to show the mantle structure. Iso-surfaces >4.35 km/s are also shown as contrasting structures in the uppermost mantle. Abbreviations are the same as in Fig. 2.

Our newly-constructed 3-D V_S_ model from ambient noise tomography shows significant lateral variations of crust and uppermost mantle down to 120-km depth from the southern to the northern Tibetan Plateau. At shallow depth, the velocity model shows low-V_S_ regions corresponding to the main sedimentary basins, i.e. Qaidam Basin and Hoh-Xil Basin (Fig. 2a). The most intriguing feature of the 3-D V_S_ model is the low-V_S_ (<3.2 km/s) anomalies in the middle crust (20–40-km depth, Figs 2 and 3) occupying almost the whole Tibetan Plateau except the Qaidam Basin. The widespread low-V_S_ anomalies within the middle crust imaged here generally agree with previous V_S_ models based on surface waves in the Tibetan Plateau [13,28,29] but show much more detail, especially the lateral connectivity of the crustal low velocity zones (Fig. S10). The crustal low-V_S_ anomalies in our 3-D V_S_ model are extensively located in the Hoh-Xil Basin, forming a continuous ∼300-km wide and W–E oriented LVZ in the northern Tibetan Plateau (named NCL in Figs 2 and 3). In the southern Tibetan Plateau, the low-V_S_ anomalies are clearly isolated into several LVZs (named SCLs in Figs 2 and 3) and separated from the NCL. Although SCLs are in smaller and weaker anomalies compared to the NCL, they are reliable anomalies as indicated by checkerboard tests (Fig. S8). Synthetic tests for connected and segmented mid-crustal low-V_S_ anomalies indicate that our result can reliably image the lateral connectivity of crustal LVZs in the Tibetan Plateau (Fig. S9). The lowermost crust is more homogeneous again (60-km depth, Fig. 2c); we are already observing mantle velocities below the Qaidam Basin, which is in agreement with the relatively thinner crust of ∼50-km thick [3,4,30].

The observed crustal LVZs in our 3-D V_S_ model show interesting correlations with the low-V_S_ anomalies in the uppermost mantle. The strongest mantle low-V_S_ anomaly at a depth of 100 km is mainly located beneath the western Hoh-Xil Basin (Fig. 2d), where our 3-D V_S_ model exhibits >10% velocity reduction compared to the typical V_S_ of ∼4.5 km/s in the uppermost mantle [31]. This strongest low-V_S_ anomaly extends to the east in a narrow zone (named NML in Figs 2 and 3) along the Hoh-Xil Basin with slightly decreased amplitude, which is almost collocated with the NCL in the northern Tibetan Plateau (Figs 2 and 3 and Fig. S11). The NML is located in the center of the area where the mantle lithosphere is the thinnest [32–34] and roughly corresponds to the core region of inefficient high-frequency S_n_ propagation observed in North Tibet [18]. In contrast, the upper mantle in the southern Tibetan Plateau mainly shows relatively high V_S_ with only few localized low-V_S_ anomalies. Such north–south different mantle velocity structures have also been similarly constrained by traditional seismic tomography [20,28] and full waveform inversion [19] (Fig. S11). Moreover, the localized N-S elongated low-V_S_ anomaly in the Lhasa Terrane at 90°E (named SML in Figs 2 and 3) agrees with the elongated LVZ shown in previous tomographic models based on both body wave [20,35] and surface wave [28]. The consistency between our 3-D V_S_ model and previous studies suggests that the NML and SML are real features (Fig. S11), although surface wave dispersions extracted from ambient noise interferometry have a more limited resolution at the mantle depth than in the crust.

## DISCUSSION

### Characteristics of crustal melting in the Tibetan Plateau

Widespread low-V_S_ anomalies in the middle crust (20–40-km depth) with different characteristics across the Tibetan Plateau are key observations from our 3-D V_S_ model. The thermal regime and material state of the crust identified from such peculiar V_S_ structures have geologic implications for the plateau evolution. Post-collisional magmatic rocks and xenoliths from the Hoh-Xil Basin, where the connective NCL imaged, indicate that the crust at 20–50 km depth is mainly constructed from metasedimentary and intermediate-mafic granulites with a SiO_2_ content of 50–70 wt% [15,25]. Phase equilibrium modeling [36] and laboratory measurements [37] indicate that the V_S_ of such a crustal composition should not be lower than 3.5 km/s at a depth of 20–50 km, which is ∼10% higher than the 3.2-km/s V_S_ observed in the crustal LVZs in this study. Our 3-D V_S_ model is based on Rayleigh waves and therefore on vertically polarized V_S_, which is subject to radial anisotropy. Isotropic V_S_ may be different than that inferred from inversion of Rayleigh wave dispersion if radial anisotropy exists in the crust, and positive radial anisotropy higher than 5% has been observed at a depth of 25–35 km in the northern Tibetan Plateau [38,39]. Hacker *et al*. [36] proposed that the vertically polarized V_S_ at a depth of 25–35 km can be reduced but not less than 3.35 km/s due to the positive radial anisotropy in the Tibetan crust. Therefore, V_S_ less than ∼3.35 km/s in the middle-lower crust implies the presence of partial melting, and we choose it as the reference V_S_ to estimate the melt fractions at the depth of 30 km.

**Figure 4.**
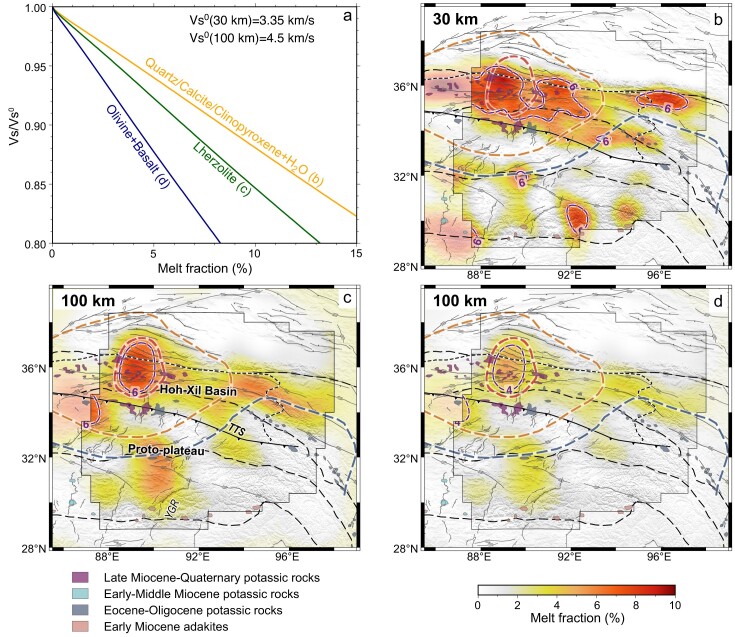
Partial melting in the crust and uppermost mantle of the Tibetan Plateau. (a) The relationship between V_S_ and melt fraction for different mineralogies [40,41]. The quartz/calcite/clinopyroxene + water system is relevant for the middle crust. Lherzolite and olivine + basalt systems represent plausible endmember mantle mineralogies. (b) Melt fractions at 30-km depth calculated from the V_S_-melt fraction relationship for the quartz/calcite/clinopyroxene + water system and the reference V_S_^0^ of ∼3.35 km/s. (c) Melt fractions at 100-km depth calculated from the V_S_-melt fraction relationship for the Lherzolite system and the reference V_S_^0^ of ∼4.5 km/s. (d) Same as (c) but for the olivine + basalt system.

Following the V_S_-melt fraction relationship of the crustal mineral system [40,41] (Fig. 4a), our 3-D V_S_ model indicates widespread partial crustal melting in the northern Tibetan Plateau (Fig. 4b). At 30-km depth, a melt fraction of at least ∼8% exists beneath the western Hoh-Xil Basin. Young felsic volcanic rocks exposed there in widely dispersed outcrops since the Pliocene also suggest high temperatures of 700–1150°C at 15–50-km depth and confirm the presence of partial melting in the middle-lower crust [14,15,25,26]. Beneath the partially molten crust, our 3-D V_S_ model exhibits a widespread uppermost mantle low-V_S_ anomaly (NML) (Figs 2 and 3). This feature is also shown in previous seismic velocity models from multiple methods [19,20,28] and corresponds approximately to the zone of inefficient high-frequency S_n_ propagation in the northern Tibetan Plateau [18]. In our model, the NML is confined in the western Hoh-Xil Basin, where a presence of ∼4%–6% melts in the uppermost mantle is estimated from the V_S_ at 100-km depth (Fig. 4c and d) and is inferred in the center of the thinnest mantle lithosphere [32–34]. The partially molten uppermost mantle spatially coincides with exposed Late Miocene–Quaternary potassic rocks in North Tibet [16,17], whose geochemical characteristics suggest a primitive source mostly derived from the partial melting of the lithospheric mantle [42]. Therefore, widespread LVZs spatially correlated in the crust and uppermost mantle highlight the important role of mantle melting and its induced magmatism in constructing the partially molten crust in the northern Tibetan Plateau.

In the southern Tibetan Plateau, melt fractions of the crust and uppermost mantle are significantly decreased compared to the north (Fig. 4b–d). Our model indicates sparsely distributed pockets of partial melting in the middle crust (Fig. 4b). We test different reference V_S_ in the estimation of melt fraction to assess the effect of crustal heterogeneity on the result, and find that the melting degree of the partially molten crust does have some uncertainty but the pattern of melt distribution is reliable (Fig. S12). The pattern of sparsely distributed melts is also shown in the 3-D electrical resistivity model of the southern Tibetan Plateau [43]. Crustal partial melting in the southern Tibetan Plateau is mostly found along several north–south-trending rifts associated with post-collisional magmatism [16,17], so the localized mantle upwelling beneath these rifts was proposed to form the isolated partially molten crust [43–45]. However, only the crustal melting along the Yadong-Gulu Rift (YGR) is observed to be associated with a weak mantle melting (Fig. 4b–d), indicating the effect of mantle dynamics should be limited. The felsic crust has relatively high radioactive heat production and can be melted by itself if thickened enough [46,47]. Localized foundering of the thickened mafic crust has been suggested to have occurred in the southern Tibetan Plateau [48] and could have left behind a thick felsic crust [49,50]. Hence, localized crustal foundering [48] and small-scale mantle dynamics [43–45] might together lead to the middle crust in the southern Tibetan Plateau being partially melting in some small areas.

### Northward growth of the proto-plateau

With the India-Asia convergence entering the ‘hard collision’ phase at ∼45 Ma [16,22], the collision stress could effectively be transferred through the proto-plateau to further north and drive the shortening and uplift of the Hoh-Xil Basin [22,24] (Fig. 1b). However, the crust beneath the Hoh-Xil Basin was only tectonically shortened for about a quarter in the Cenozoic according to surface deformation, which cannot alone produce the almost doubled crustal thickness there [24]. Thus, additional processes are necessary for the northward growth of the proto-plateau.

The underthrusting of the Indian slab beneath the Tibetan Plateau [51–54] strongly influences the lithospheric thermal state of the proto-plateau. The fragmentation and detachment of the subducted Indian slab has been suggested to generate adakitic and potassic rocks scattered in the southern Lhasa Terrane in the Late Oligocene–Miocene [16,17,54–56]. Our 3-D V_S_ model images the SML nearly at 90°E beneath the north of the YGR (Fig. 2), this area is also characterized by small azimuthal anisotropy as determined from shear wave splitting (Fig. S11c). Geophysical evidence suggests that the underlying Indian slab is segmented across the YGR [20,35,57]. The Indian slab is steeply subducting to the west of the YGR [52,58,59] (Fig. S11c), whereas to the east it is underthrusting sub-horizontally and reaching further north [20,60,61]. The Indian slab tear could have driven small-scale upwelling mantle flow [20,35]. Except for the thermal state variation in the crust and uppermost mantle attributed to this small-scale mantle upwelling, the lithosphere of the southern Tibetan Plateau was overall kept away from modification due to the protection of the underlying Indian slab, according to the relatively high V_S_ in the uppermost mantle constrained by this study (Figs 2 and 3) and previous models [19,20,28]. The cessation of mantle-derived magmatism in the southern Lhasa Terrane after the Late Miocene also supports that the underthrusting Indian slab progressively shut off the heat from the asthenosphere [16,17,19,22] (Fig. 1b). Although a crust with a thick felsic layer could also be heated by itself due to radioactive heating [46,47], such felsic crust was only reported in some areas with localized crustal foundering [48,49]. Consequently, partially molten crust only exists in some isolated areas in the proto-plateau after the crust was shut off from basal heating (Fig. 4b). These isolated areas with crustal partial melting in the proto-plateau are less connected to the widespread partially molten crust in the north (Fig. 4b), implying that the partially molten crust in the Hoh-Xil Basin would not be continuously transported from the proto-plateau. The azimuthal anisotropy in the crust with mainly E–W fast-propagation direction also denies a northward crustal channel flow in this area [62,63]. Therefore, the lithospheric thermal state revealed from our 3-D V_S_ model indicates that the proto-plateau could not have grown incrementally to the north via crustal channel flow and promoted crustal thickening in the Hoh-Xil Basin.

In the northern Tibetan Plateau, with no intervening Indian slab, the thickened lithosphere could be delaminated or thermally eroded owing to gravitational instability and induce mantle upwelling to isostatically support further uplift since the Miocene [11] (Fig. 1b), which can be inferred by significantly thinned lithosphere [32–34] and the evidence for a detached remnant in the mantle transition zone [64]. The good spatial correlation between the strong NML imaged in this study and Late Miocene–Quaternary mantle-derived potassic magmatism in the Hoh-Xil Basin further supports the hypothesis of lithospheric root removal and mantle upwelling [16,19,42]. Meanwhile, the hot and melting uppermost mantle could contribute to the thickening of the crust via magmatic underplating and subsequent magmatic intrusion within the crust [26]. Our improved 3-D V_S_ model benefiting from direct seismic observations in the Hoh-Xil Basin shows a widespread NCL collocated with the NML (Figs 2 and 3), which indicates the partially melted crust heated by large-scale asthenosphere upwelling and mantle-derived magmatism. In the last few million years, small-volume magmas deeply derived from crustal melts erupted in the Hoh-Xil Basin [14,15,25,26], supporting that the present Hoh-Xil Basin has a thick crust heated by the hot mantle and magmatic intrusion. Moreover, our results show that the highest partial melting degree in both the middle crust and the uppermost mantle is located in the same spot beneath the western Hoh-Xil Basin (Fig. 4b–d). Given the westward younger trend of the post-collisional magmatism there [16,17] (Fig. 1a), the correlated crustal and mantle melting beneath the western Hoh-Xil Basin indicates that a possible westward propagating pattern of lithospheric root removal and magmatic intrusion might have controlled the thermal evolution and crustal growth in the northern Tibetan Plateau since the Miocene [34,65]. Widespread crustal partial melting generated in this process could have reduced the strength of the middle crust [66] to form a uniform elevation in the northern Tibetan Plateau as proposed in the classic channel-flow hypothesis [10].

**Figure 5.**
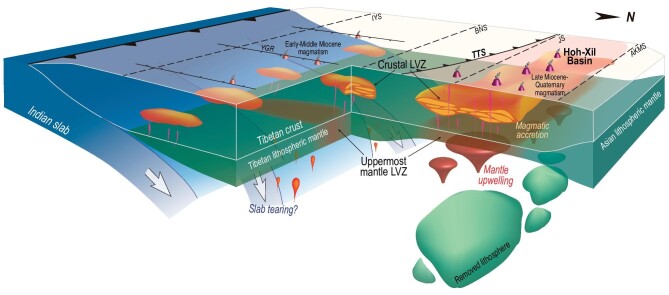
Geodynamic interpretation of Tibetan Plateau growth. Modification of the lithosphere of the southern Tibetan Plateau had been mainly prevented due to the protection of the underlying Indian slab since the Miocene. With no intervening Indian slab, the thickened lithosphere in the Hoh-Xil Basin was delaminated or thermally eroded to induce mantle upwelling and magmatic intrusion in the crust, which are featured by the spatial correlation of extensive melting in the middle crust and uppermost mantle with young crust-/mantle-derived magmatism in the Hoh-Xil Basin. The magmatic accretion and corresponding melting have thickened the crust and reduced the crustal strength to form a uniform elevation, thus promoting the northward growth of the plateau.

Therefore, collocated crustal and uppermost mantle LVZs imaged in our 3-D V_S_ model suggest a relationship between the partially molten crust and mantle dynamics in the Hoh-Xil Basin. We interpret the uplift of the Hoh-Xil Basin as a result of lithospheric mantle delamination which induced mantle upwelling and magmatic intrusion to thicken the crust and promote the northward growth of the proto-plateau (Fig. 5). Similar magmatic accretion related to lithospheric root removal also has been suggested to cause pre-Miocene crustal thickening in the southern Tibetan Plateau [22,67,68]. These findings thus lead to critical implications for the role of delamination-driven magmatism in the continental crust growth in collision orogens.

## CONCLUSIONS

Using recently available data recorded by seismic arrays located in the Hoh-Xil Basin, we construct a 3-D V_S_ model of the Tibetan Plateau by ambient noise tomography. We observe widespread and correlated low-V_S_ anomalies in the crust and uppermost mantle beneath the Hoh-Xil Basin with an unprecedented resolution, which cannot be explained by thermal or compositional effects and therefore require the presence of melts. Their spatial coincidence with exposed young magmatic rocks suggests that the Hoh-Xil Basin was uplifted by lithospheric mantle removal and subsequent magmatic intrusion. Considering the Hoh-Xil Basin had remained a foreland basin adjacent to the proto-plateau until the Early Miocene, the mechanism of its rapid uplift revealed in this study implies that the lithosphere delamination and its driven magmatism promote the growth of the Tibetan Plateau.

## DATA AND METHODS

The data analyzed in this study are mainly from 226 broadband seismic stations linearly deployed across the northern Tibetan Plateau with an average interstation distance of ∼15 km (Fig. 1a and Fig. S1a). These stations were operated by the Chinese Academy of Geological Sciences from 2008 to 2020. We also collect synchronous data recorded by permanent broadband seismic stations from the China National Seismic Network (CDSN) and temporary seismic stations from the INDEPTH IV network. In total, we analyzed continuous data from 363 broadband seismic stations operated from 2007 to 2020 (Figs S1 and S2) to extract Rayleigh wave signals from ambient noise. We pick group and phase velocity dispersions of Rayleigh wave by using the Automatic Frequency-Time ANalysis (AFTAN) package. We finally obtained more than 13 000 group and phase velocity dispersion curves (Fig. S3c), which cover the study region well (Fig. S4). In this study, we apply the direct inversion method [69] to construct the 3-D V_S_ model without the intermediate step of inverting phase or group velocity maps. Combining the theoretical seismic velocity-contiguity relationships [40] and the experimental contiguity-melt fraction relationships for variable systems [41], we estimate the melt fractions of crust and uppermost mantle in the Tibetan Plateau from the 3-D V_S_ model (Fig. 4). The Supplementary Data provides more details of the inversion of the 3-D V_S_ model and the estimation of crustal and mantle melt fractions.

## Supplementary Material

nwae257_Supplemental_File
