# Supplementary material for: Correlated crustal and mantle melting documents proto-Tibetan Plateau growth
Source: Natl Sci Rev. 2024 Jul 26;11(9):nwae257. doi: 10.1093/nsr/nwae257 (PMC11376070; doi:10.1093/nsr/nwae257)
Supplement: nwae257_Supplemental_File [file nwae257_supplemental_file.pdf]

Supplementary Data for

Correlated crustal and mantle melting documents proto-Tibetan Plateau growth

Wei Li[1,2], Rizheng He[3,*], Xiaohui Yuan[2,*], Felix Schneider[2], Frederik Tilmann[2,4], Zhen Guo[5], Yongshun John Chen[5]

[1] School of Geophysics and Geomatics, China University of Geosciences, Wuhan 430074, China
[2] Deutsches GeoForschungsZentrum GFZ, Potsdam 14473, Germany
[3] SinoProbe Laboratory, Chinese Academy of Geological Sciences, Beijing 100094, China
[4] Freie Universität Berlin, Berlin 12249, Germany
[5] Department of Ocean Science and Engineering, Southern University of Science and Technology, Shenzhen 518055, China
[*]Corresponding author, E-mail: herizheng@cags.ac.cn, yuan@gfz-potsdam.de

Contents of this file

Supplementary Data and Methods
Supplementary Figures 1 to 12
Supplementary References

Supplementary Data and Methods

Data processing and dispersion analysis

The data analyzed in this study are collected from 363 broadband seismic stations operated from 2007 to 2020 (Supplementary Figs. 1 and 2). Temporary seismic stations are mainly from 226 broadband seismic stations linearly deployed across the northern Tibetan Plateau with an average interstation distance of ~15 km (Fig. 1a and Supplementary Fig. 1a). These stations were operated by the Chinese Academy of Geological Sciences from 2008 to 2020, including subnetworks NT_QT (70 stations), NT_GZ (16 stations), NT_SN (40 stations), NT_BLJ (31 stations), and NT_TQ (70 stations). We also collected 76 temporary seismic stations from the INDEPTH IV network [1] operated from 2007 to 2009 with an average interstation distance of

~50 km (Fig. 1a and Supplementary Fig. 1a). As deployment periods of the temporary seismic networks used in this study did not substantially overlap in time, surface wave signals at long periods extracted from ambient noise interferometry would be limited by the short interstation distances within each sub-network, and consequent loss of resolution in the deep crust and mantle. Thus, to improve the data coverage in the northern Tibetan Plateau, we also processed 38 permanent broadband seismic stations from the China National Seismic Network (CDSN) which overlap in time with almost all temporary networks (Supplementary Figs. 1a and 2).

We extract Rayleigh wave signals from ambient noise similarly to previous studies [2,3]. After removing the instrument response, resampling (1 Hz), low-pass filtering (corner 0.01 Hz), applying time-domain normalization and spectral whitening, we calculate cross-correlations for all simultaneously recorded station pairs using 1-hour length segments. The hourly cross-correlations in a day are saved and linearly stacked to a daily one. Then, we average the positive and negative lags and linearly stack all daily cross-correlations over the entire operating period. In total, we obtain stacked cross-correlations for more than 22000 station pairs. Significant surface wave signals with velocities of 2–4 km/s can be recognized in the stacked cross-correlations at different periods (Supplementary Fig. 1b).

We pick group and phase velocity dispersions of Rayleigh wave by using the Automatic Frequency-Time ANalysis (AFTAN) package. After this automatic procedure, we manually checked all dispersion curves and retained dispersions that are continuous over a wide range of periods. We also impose a minimum interstation distance control following previous studies [2,3], and the threshold is set to be greater than two wavelengths. For both group velocity and phase velocity, we finally obtained more than 13000 dispersion curves (Supplementary Fig. 3c). More than 10000 dispersion measurements are available at each period between 8–30 s, decrease gradually in the longer (35–65) periods. Less than 1500 valid measurements are available at long periods of 70–100 s (Supplementary Fig. 3c). Thus, we only use measurements in the periods of 6–65 s in the inversion of 3-D Vs model. These group and phase velocity measurements cover the study region well (Supplementary Fig. 4). At short periods, the measurements along ray paths across basins, i.e., Qaidam Basin in the northeastern Tibetan Plateau and Hoh-Xil Basin in North Tibet, show significantly low velocities. At long periods, the velocity is high across the Qaidam Basin but still low across the Hoh-Xil Basin.

Direct inversion of 3-D V_S model

We apply the direct inversion method [4] to construct the three-dimensional (3-D) V_S model. The key to the inversion problem is to seek a model that minimizes the travel time residuals, that is, the deviation of the observed travel times ($\boldsymbol{d}$) from the ones calculated from the model ($\boldsymbol{m}$).

Accordingly, this can be expressed in the classical matrix form $\boldsymbol{d} = \boldsymbol{Gm}$ and can be solved by several iterations to minimize the objective function as following:

$$\Phi(m) = \|\boldsymbol{d} - \boldsymbol{Gm}\|_2^2 + \lambda\|\boldsymbol{Lm}\|_2^2$$

where $\boldsymbol{L}$ is a model smoothing operator, which is set to the second order of the spatial derivative operator, and λ is the weighting parameter balancing data residual term $\|\boldsymbol{d} - \boldsymbol{Gm}\|_2^2$ and model regularization $\|\boldsymbol{Lm}\|_2^2$. The sensitivity matrix $\boldsymbol{G}$ can be calculated from the initial model, which is updated after each iteration.

Here, we mesh the study region as 24-layer grids with the depth interval of 5 km at 0–80-km depth, 10 km at 90–100-km depth, 15 km at 115–145-km depth, and 20 km at 160–180-km depth (Supplementary Fig. 5c). We set the V_S in the initial model as the 1-D Vs model inverted from the average group and phase velocity dispersion curves (Supplementary Figs. 3a and 3b) using the Computer Programs in Seismology package [5] (Supplementary Fig. 5c). The meshed grids have horizontal intervals of 0.4 ° in latitude and longitude, which was chosen based on available ray coverage (Supplementary Fig. 5d) and tests with different grid sizes (Supplementary Fig. 6a). We can find a decrease of the residual when the grid size decreases from 0.5 ° to 0.4 °, but the residual decreases only slightly when the grid size further decreases to 0.3 ° and even increases when using large weighting parameters (Supplementary Fig. 6a). Different weighting parameter (λ) were also tested and 70 is chosen for the preferred model, as it provides an optimal trade-off between model norm and data residual term (Supplementary Fig. 6a). With our meshed space and preferred weighting parameter, the group and phase travel time residuals show a Gaussian type distribution centered near zero after 10 iterations (Supplementary Fig. 6b). We compare the inversion with the optimal weighting parameter with those with smaller or larger weighting parameters (Supplementary Figs. 6c–e). Anomaly patterns are very similar for all these cases, demonstrating that the inversion is stable with regard to reasonable variations of the weighting parameter.

Resolution tests

Bootstrap tests are adopted to estimate the uncertainty from data errors and initial model [6]. We perform the bootstrap procedure by resampling with replacement the dispersion curve set for 50 times and then inverting for velocity models according the aforementioned procedure. The initial model for each inversion is set as the V_S randomly perturbing each layer of the average 1-D model with a standard deviation of 0.15 km/s. The average values of the output Vs models are similar to the final model of this study with small uncertainties (standard deviation < 0.05 km/s) in regions with good data constraints (Supplementary Fig. 7).

We then perform a series of checkerboard tests to estimate the resolution ability of used data and method (Supplementary Fig. 8). The input models, constructed by 5% maximum perturbation relative to the average 1-D V_S model, have various scales in horizontal and separated patterns in depths. We synthesize group and phase travel times for the paths same as in the real dataset. According to the measurement error of Rayleigh wave dispersion estimated in previous studies [2], we add 1% random noise to the synthetic dataset and invert them following the aforementioned procedure. For small anomalies (0.8°), the patterns can be recovered at shallow depths (< 80 km) but with some smearing at larger depths (> 80 km). For larger anomalies (1.2° and 1.6°), the entire depth range of the model can be reasonably resolved, except some under-recovery of amplitudes at larger depths (> 80 km). Considering the coverage of ray paths (Supplementary Figs. 4 and 5d) and the uncertainty estimated in bootstrap tests (Supplementary Fig. 7), we exclude the edge region that exists smearing in checkerboard tests (Supplementary Fig. 8) in the following discussion, which is delineated by black frames in Figs. 2 and 4.

To evaluate the resolution capability of the dataset and method with respect to the connectivity of crustal LVZs in the Tibetan Plateau, we construct the synthetic tests for connected and segmented low-V_S anomalies at depths of 25–45 km (Supplementary Fig. 9). The input model is meshed onto the same grid as that in the direct inversion procedure. Then, we synthesize group and phase travel times for the paths same as in the real dataset with added 1% random noise and invert them following the same direct inversion procedure as for the real data. Generally, the absolute velocities of the output models are smoother than the inputs, and the strength of crustal low-velocity anomaly is under-recovered. However, the horizontal and in-depth distribution of LVZs can be acceptably reconstructed in the output models.

Estimating the melt fractions of crust and uppermost mantle

The direct effects of partial melting on seismic velocity have been studied over a wide range of pore shapes, and considered to be dependent on the contiguity of grain boundaries, which is a function of dihedral angle in an equilibrium geometry [7]. The contiguity of grain boundaries is thus not solely a function of the melt fraction but also strongly dependent on the composition of the melt- or fluid-bearing rock system, and was experimentally derived for various compositions [8]. Here, combining the theoretical seismic velocity-contiguity relationships [7] and the experimental contiguity-melt fraction relationships for variable systems [8], we can obtain the relationship between V_S and melt fraction to estimate the melt fractions of crust and uppermost mantle in the Tibetan Plateau (Fig. 4).

To estimate the melt fractions from the V_S-melt fraction relationship, a reference V_S at a specific depth is required. Considering the composition inferred from the xenoliths and the radial

anisotropy induced by the mica lattice, Hacker et al. [9] concluded that the lowest vertically polarized V_S is ~3.35 km/s at a depth of 25–35 km. Thus, for V_S lower than ~3.35 km/s, partial melting should be considered. Using this value as the reference V_S, we can estimate the melt fractions at the depth of 30 km which is almost the center of the crustal LVZs imaged in this study (Fig. 2). Following the V_S-melt fraction relationship of the crustal mineral system, our results imply the presence of widespread partially melted crust in North Tibet with the highest inferred melt fraction of ~8% at 30-km depth beneath the western Hoh-Xil Basin (Fig. 4b). Referring to a typical vertically polarized V_S of ~4.5 km/s in the uppermost mantle [10], we can infer that the uppermost mantle beneath the partially melted crust in North Tibet contains ~4–6% melt from the low-velocity anomalies at depth of 100 km (Figs. 4c and 4d). In contrast, in South Tibet, the partially melted crust is only sporadically distributed and with lower melt fractions. In the uppermost mantle, evidence for partial melting is only evident near the YGR (Figs. 4b–d). It should be noted that the reference V_S should be variable due to the heterogeneity in the crust and upper mantle, hence there are some uncertainties in the estimation of melt fraction when using the same reference V_S for the whole Tibetan Plateau. Here, we use different reference V_S to calculate the melt fraction at 30-km depth (Supplementary Fig. 12). The amplitude of melt fractions does vary with the reference V_S, but the patterns of partially molten crust are almost similar by using different reference V_S. These tests indicate that the partially molten crust widespread in the northern Tibetan Plateau but sparsely isolated in the south revealed in this study is a reliable pattern.

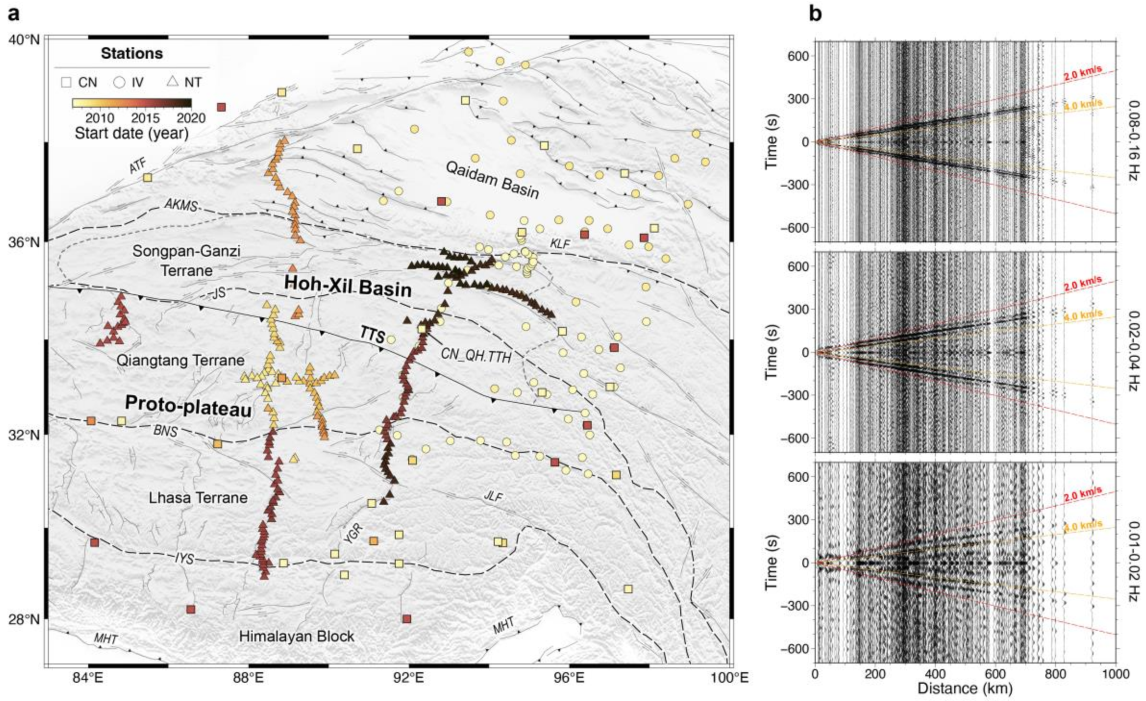

Supplementary Figure 1. (a) Maps of seismic stations from the CDSN (CN), INDEPTH-IV (IV), and temporary seismic networks in North Tibet (NT) operated by the Chinese Academy of Geological Sciences. Color indicates the start date of their operating periods. Abbreviations are the same as in Fig. 1a. **(b)** Bandpass filtered (0.08–0.16 Hz, 0.02–0.04 Hz, 0.01–0.02 Hz) cross-correlations as a function of distance and lag time for all available pairs between Station CN_QH.TTH indicated in (a) and other stations.

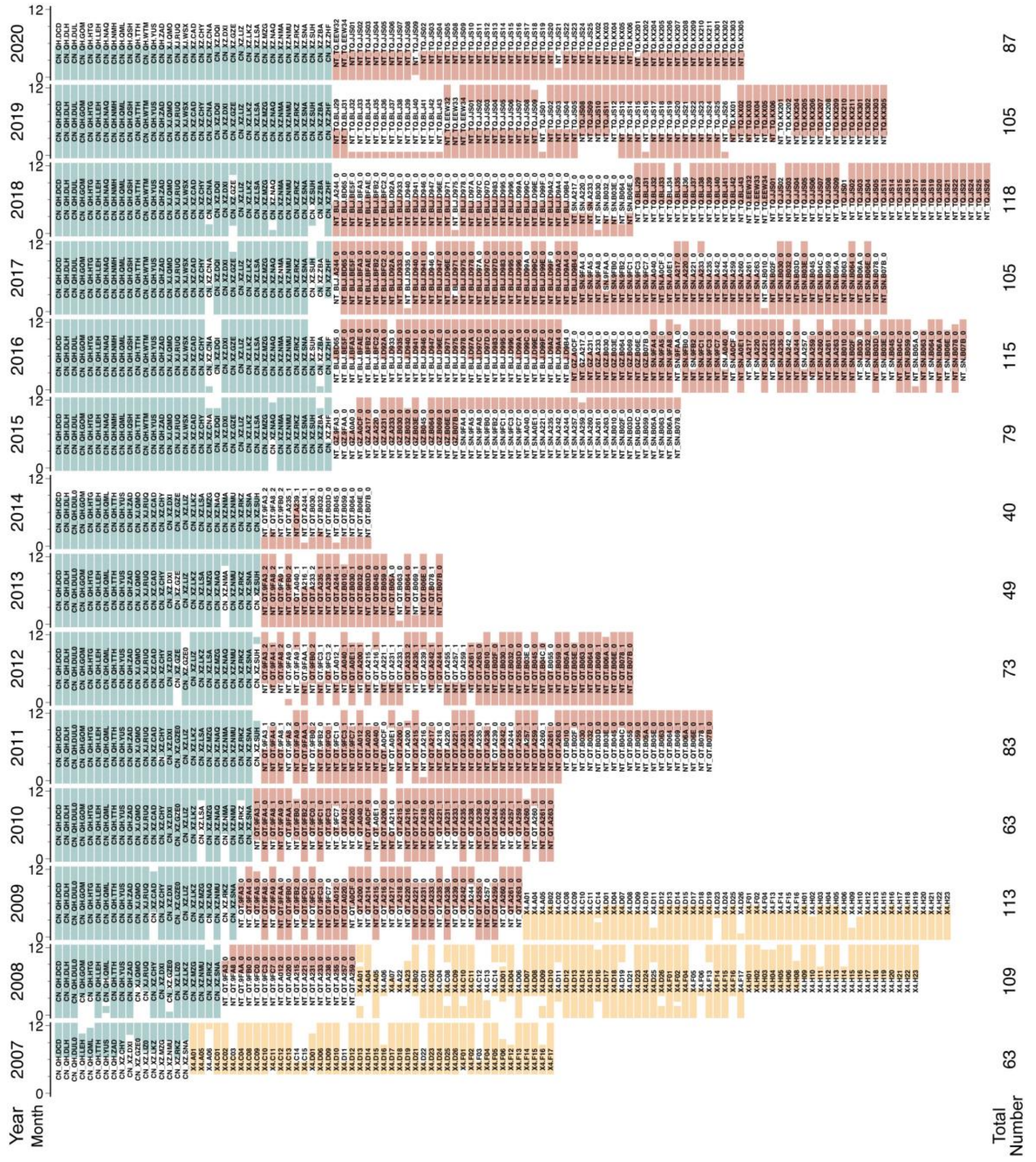

Supplementary Figure 2. Availability of data for the stations used in this study.

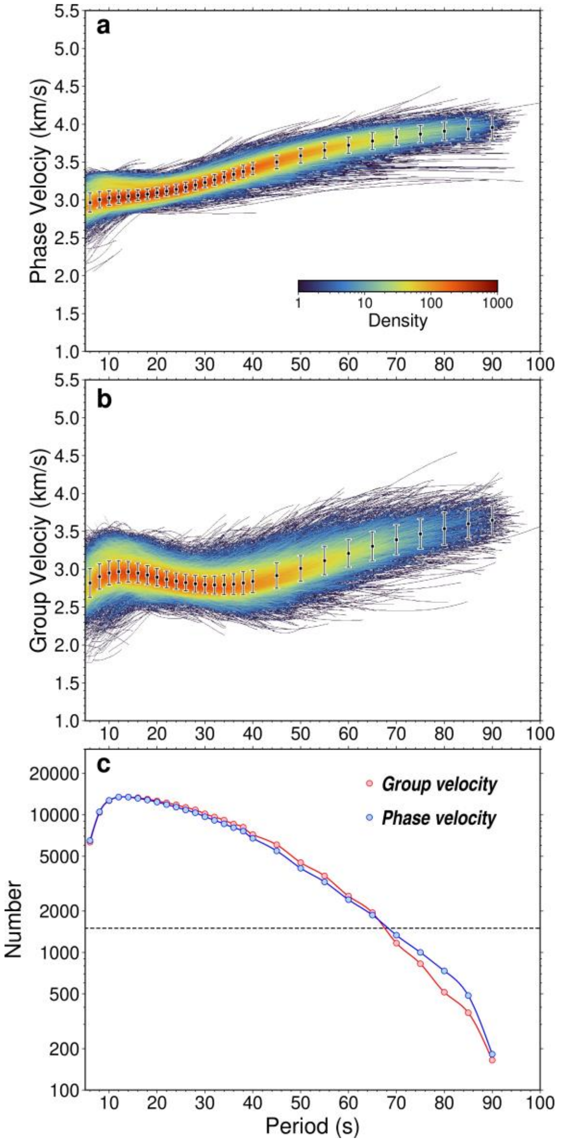

Supplementary Figure 3. (a) All picked Rayleigh wave group velocity dispersion curves. Circles show the averages at periods used in the inversion with one standard deviation indicated by the bars. **(b)** similar to (a), but for the phase velocity dispersion curves. **(c)** Numbers of Rayleigh wave group and phase velocity measurements at different periods. The dashed line marks the threshold for minimum of dispersion curves needed per period (1500), above which the measurements are used in the inversion.

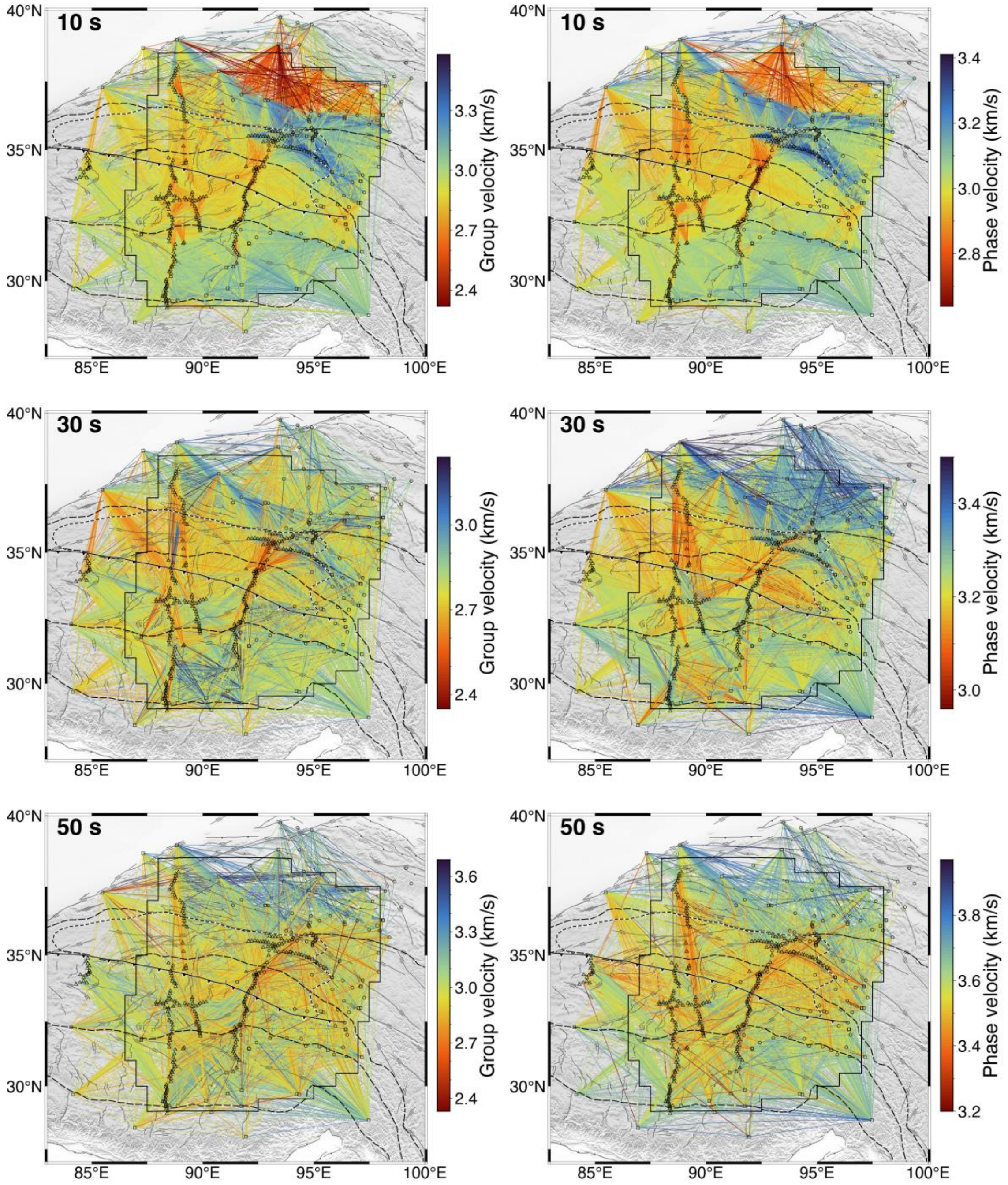

Supplementary Figure 4. Path coverages at periods of 10, 30, and 50 s for Rayleigh wave group and phase velocity measurements, which are represented along the interstation lines color-coded by corresponding velocity values. The black frame indicates the area where the final 3-D Vs model is displayed in Fig. 2.

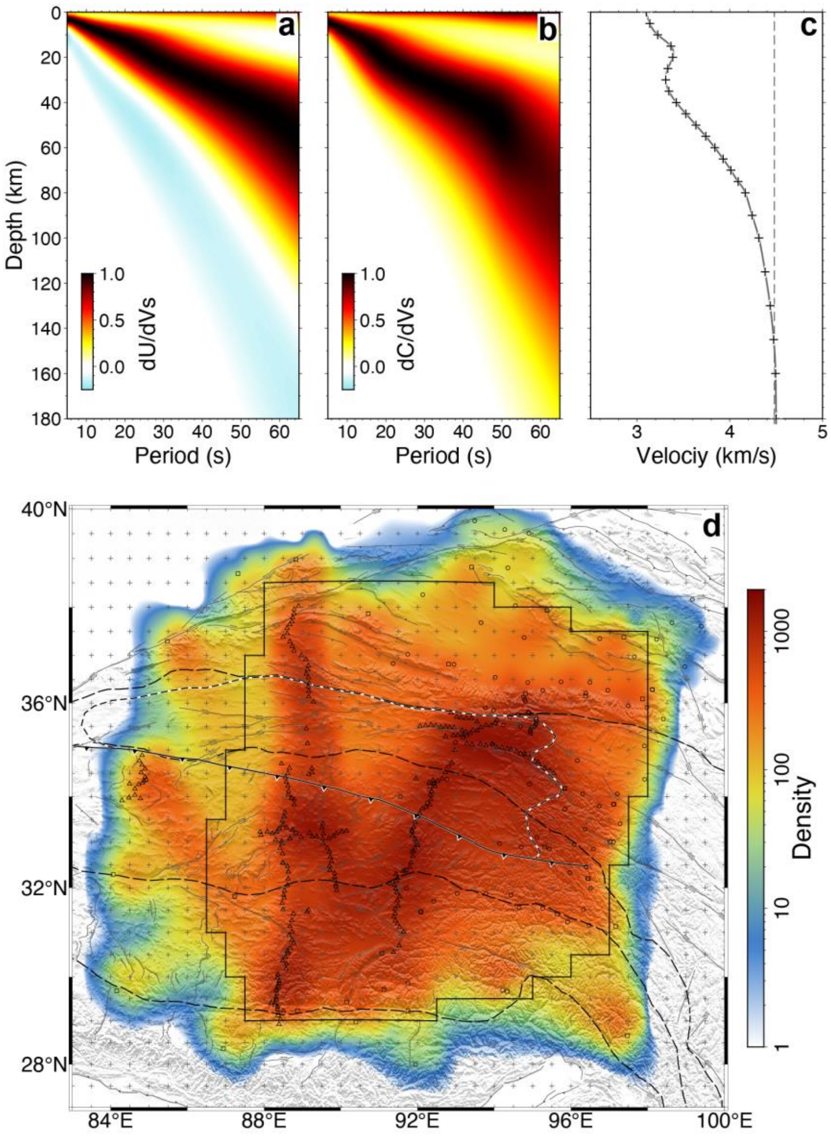

Supplementary Figure 5. (a–b) Normalized group (dU/dVs) and phase (dC/dVs) velocity sensitivity kernels of the Rayleigh wave versus periods calculated from the 1-D Vs model in (c). **(c)** 1-D Vs model inverted from average group and phase velocity dispersions shown in Supplementary Figs. 3a and 3b. Black crosses indicate 24-layer grids spacing in the depth range of 0–180 km. **(d)** Map of meshed grids (black crosses) with an interval of 0.4 ° in latitude and longitude. The background shows the hit counts of these paths. The black frame indicates the area where the final 3-D Vs model is displayed in Fig. 2.

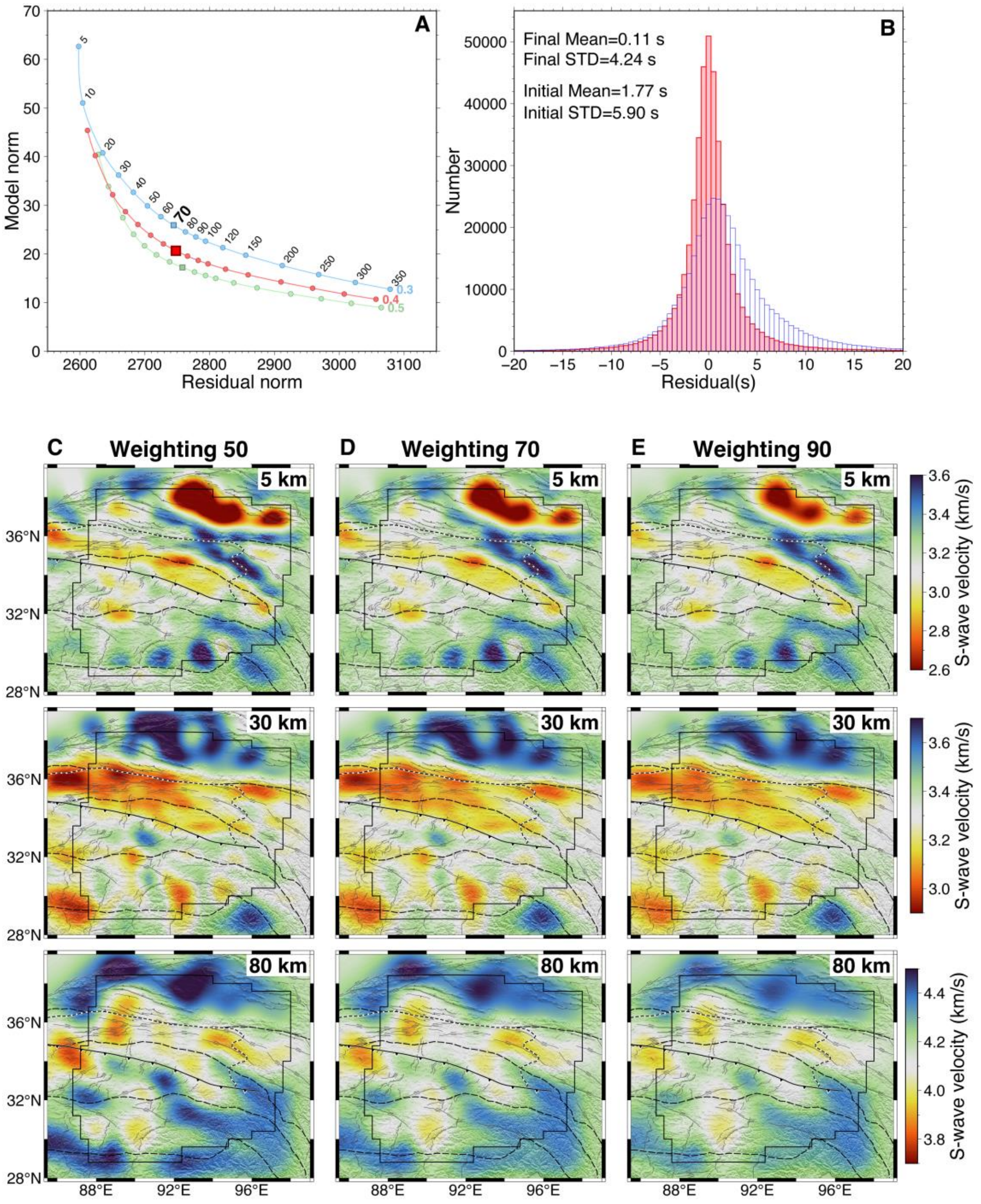

Supplementary Figure 6. (a) Trade-off between model and residual norms for different weighting parameters shown by the number beside each point and different grid sizes shown by the number beside the end of colored lines. The large red square shows the optimal weighting parameter (70) and grid size (0.4° in latitude and longitude) used in the inversion. **(b)** Distributions of travel time residuals for the initial model (blue bars) and the final model (red bars) after inversion with the optimal weighting parameter and grid size shown in (a). **(c–e)** Vs models with the grid size of 0.4° inverted with different weighting parameters of 50, 70, and 90. The black frame indicates the area where the final 3-D Vs model is displayed in Fig. 2.

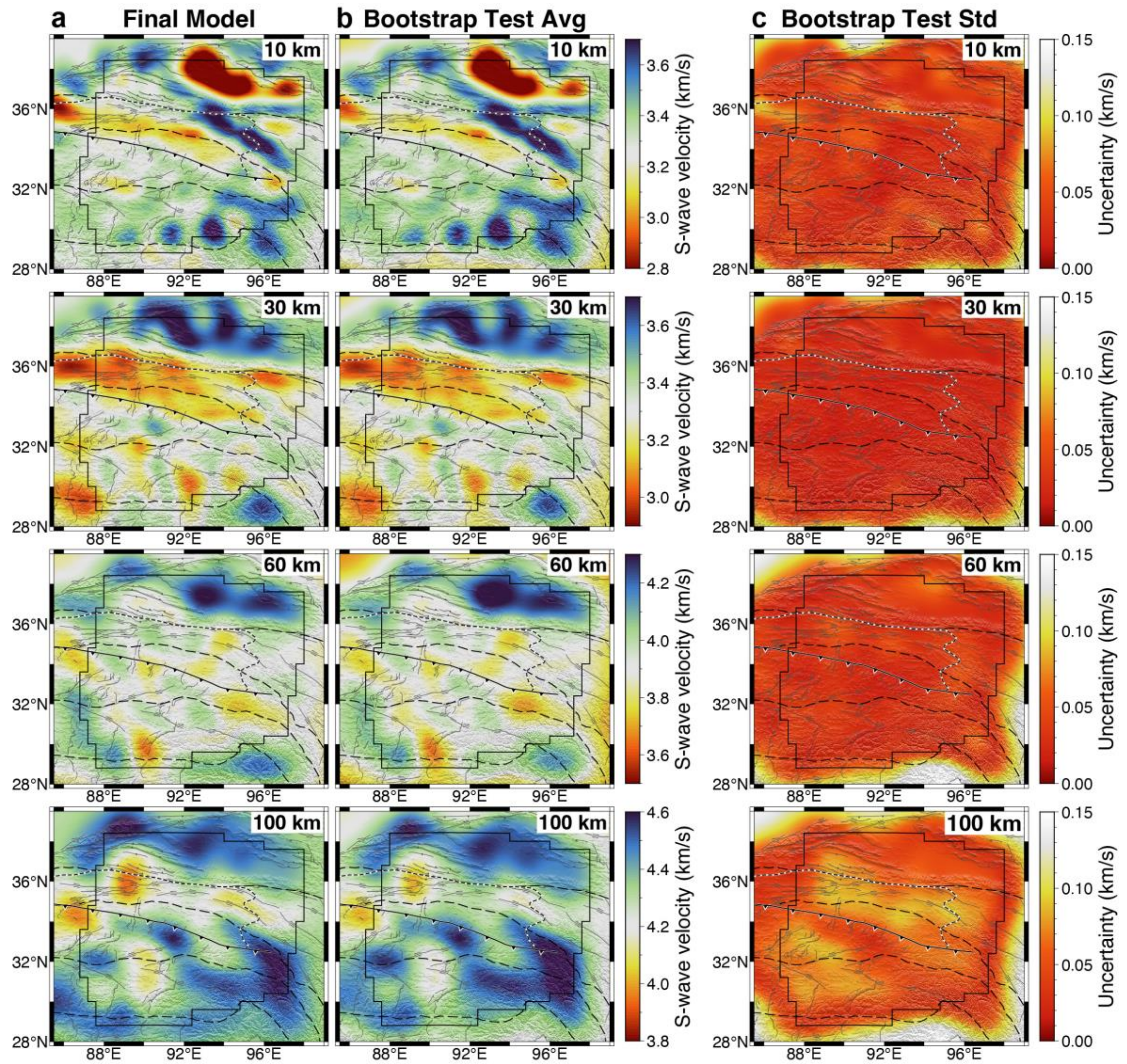

Supplementary Figure 7. Estimated uncertainties of the 3-D Vs model at depths of 5, 10, 20, and 30 km from bootstrap tests. The final 3-D Vs model obtained in this study is shown in the left panels. The middle panels show the average of the output models with standard deviations shown in the right panels. The black frame indicates the area where the final 3-D Vs model is displayed in Fig. 2.

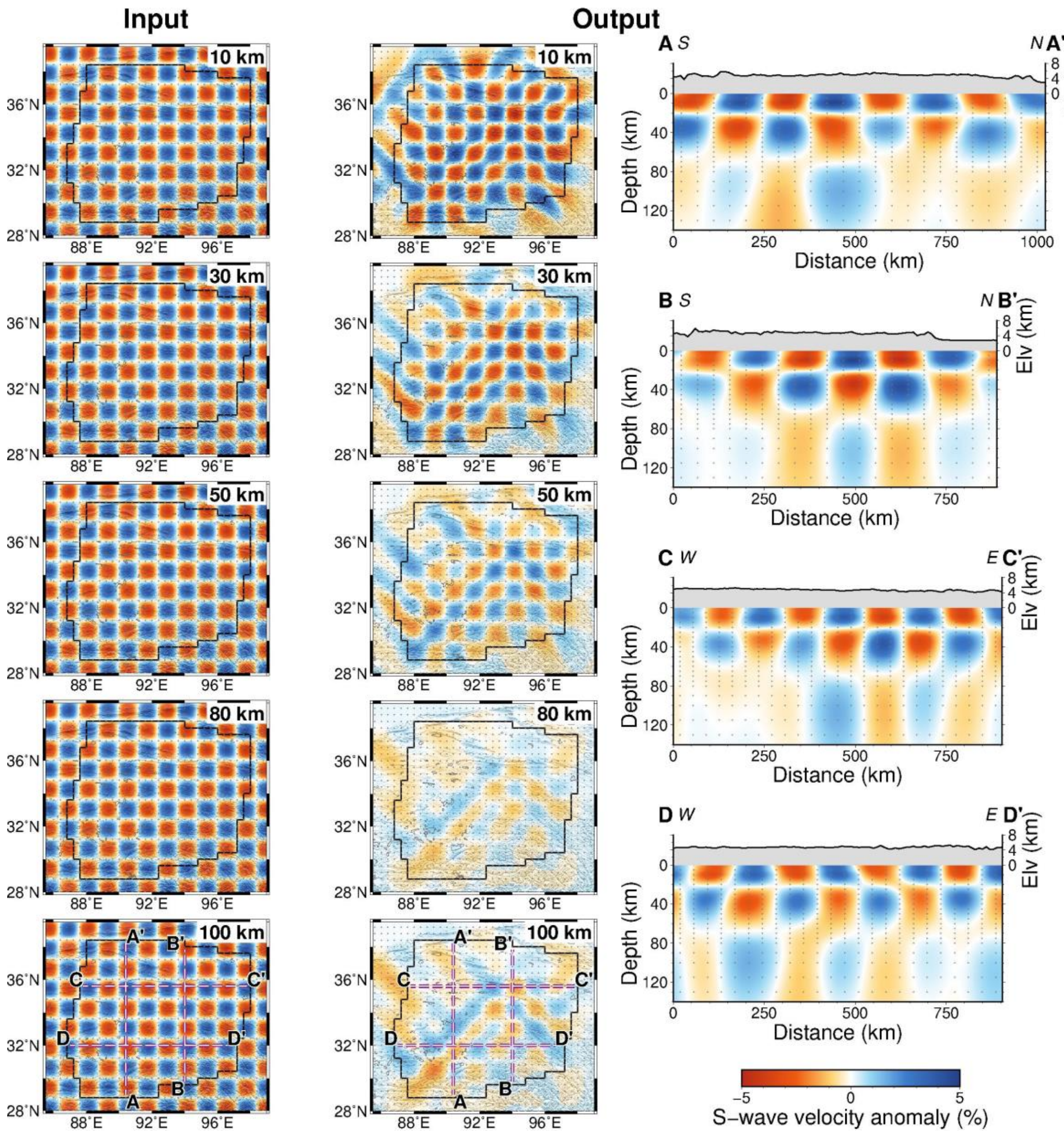

Supplementary Figure 8. Checkerboard test for anomalies with the horizontal size of checkers of 0.8°. Input model is constructed by 5% maximum perturbation relative to the average 1-D Vs model and has separated patterns for 0–20-km, 25–65-km, and 70–180-km depths. 1% random noise is added to the synthetic dataset. Black crosses indicate grid nodes. Purple dashed lines mark the location of the profiles shown in the right panels. The black frame indicates the area where the final 3-D Vs model is displayed in Fig. 2.

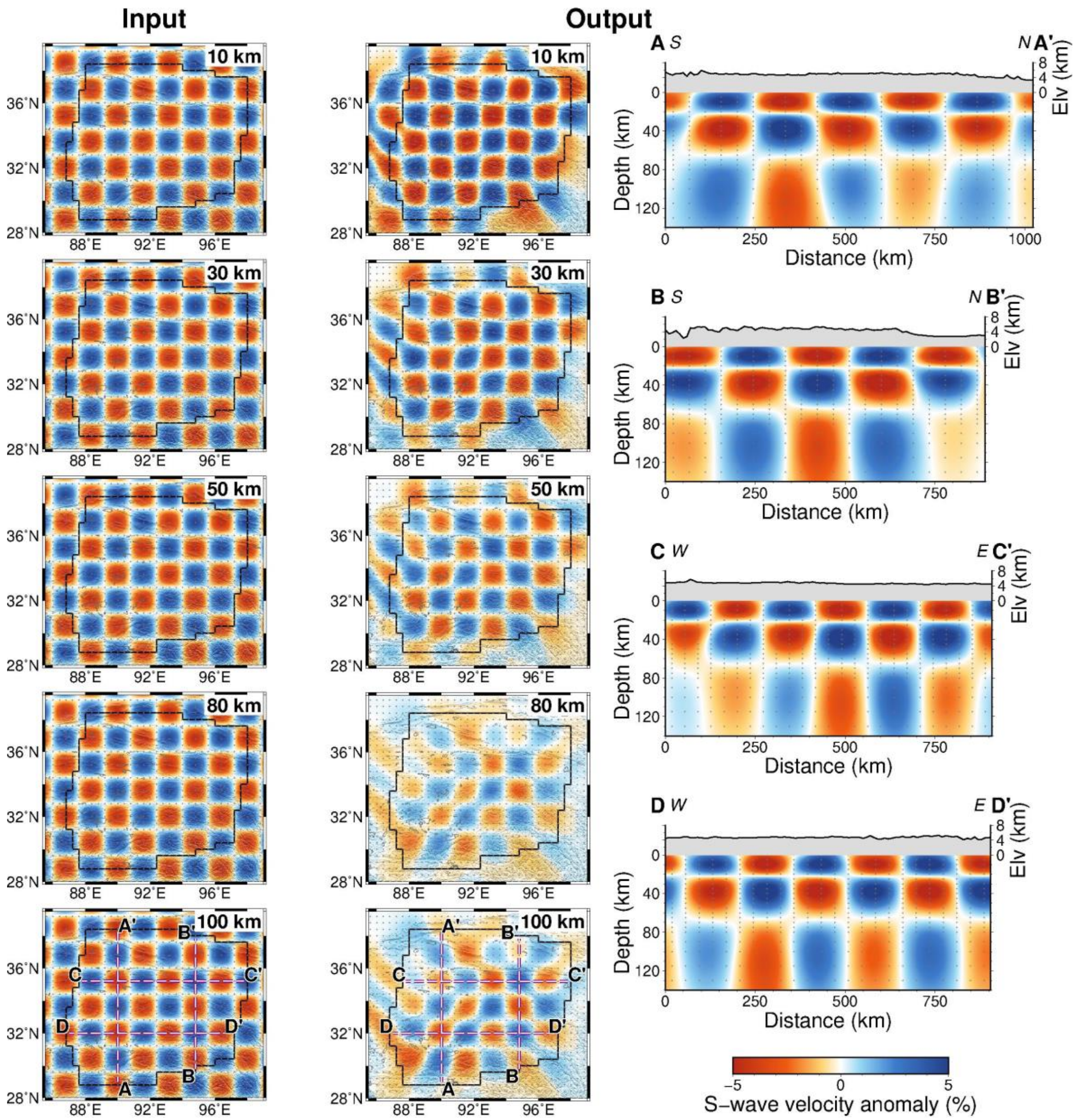

Supplementary Figure 8. Continued for anomalies with the horizontal size of checkers of 1.2 °.

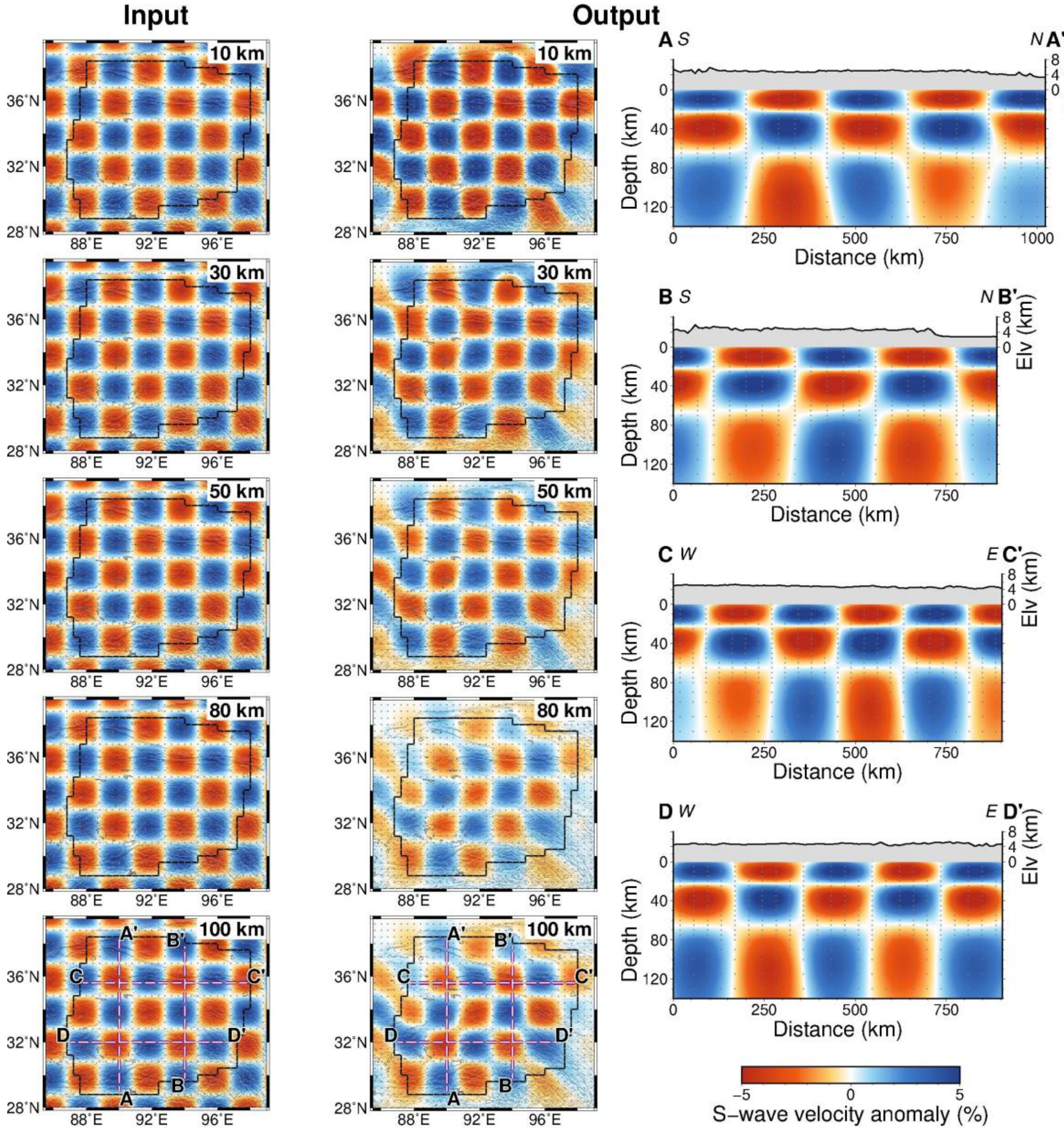

Supplementary Figure 8. Continued for anomalies with the horizontal size of checkers of 1.6 °.

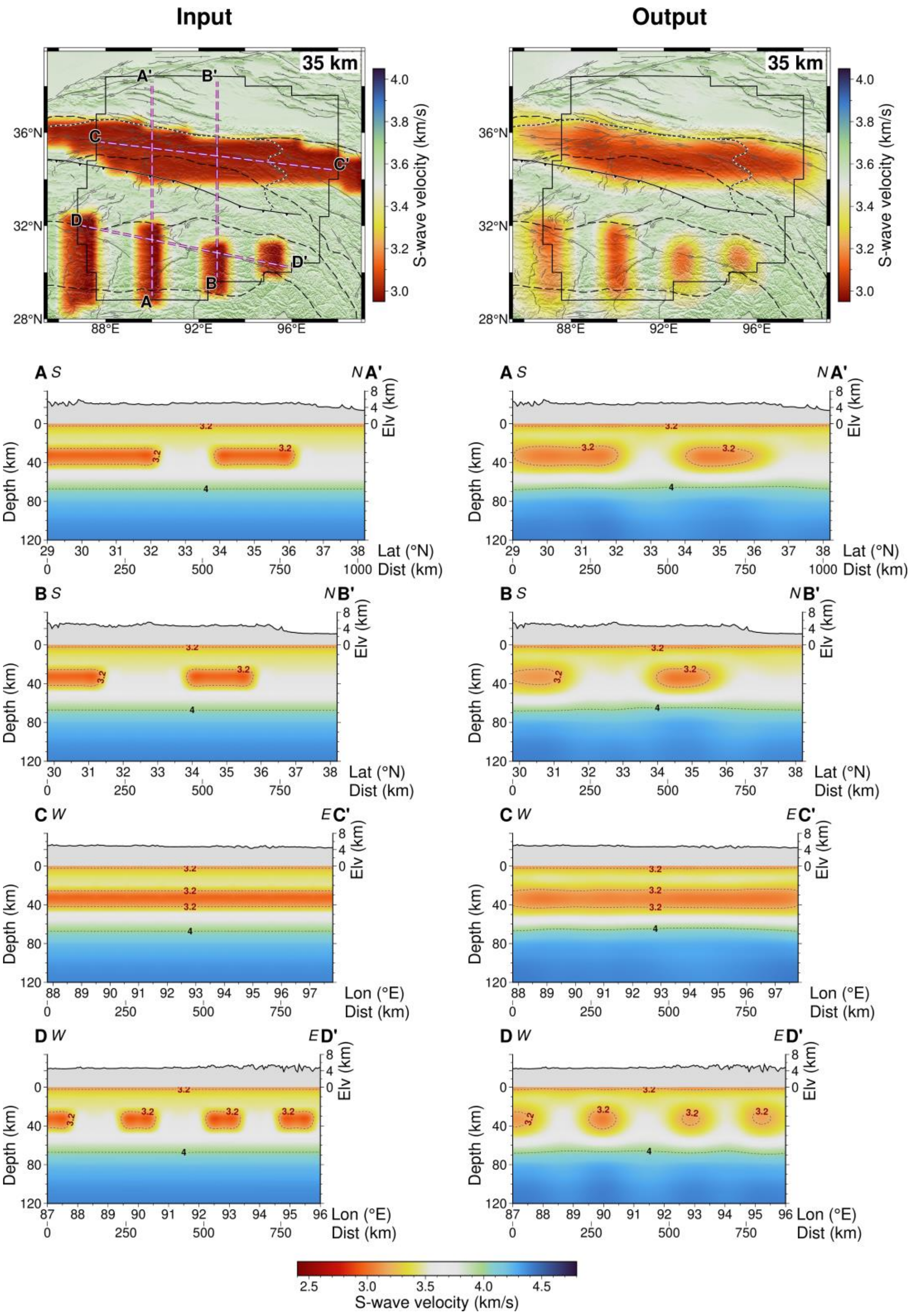

Supplementary Figure 9. Synthetic test to evaluate the resolution capability for the connectivity of crustal low-velocity zones (LVZs) at the depth of 25–45 km in the Tibetan Plateau. The input model shown in the left panel contains a widely distributed crustal LVZ in north Tibet and four separated crustal LVZs extending in north-south strips in south Tibet.

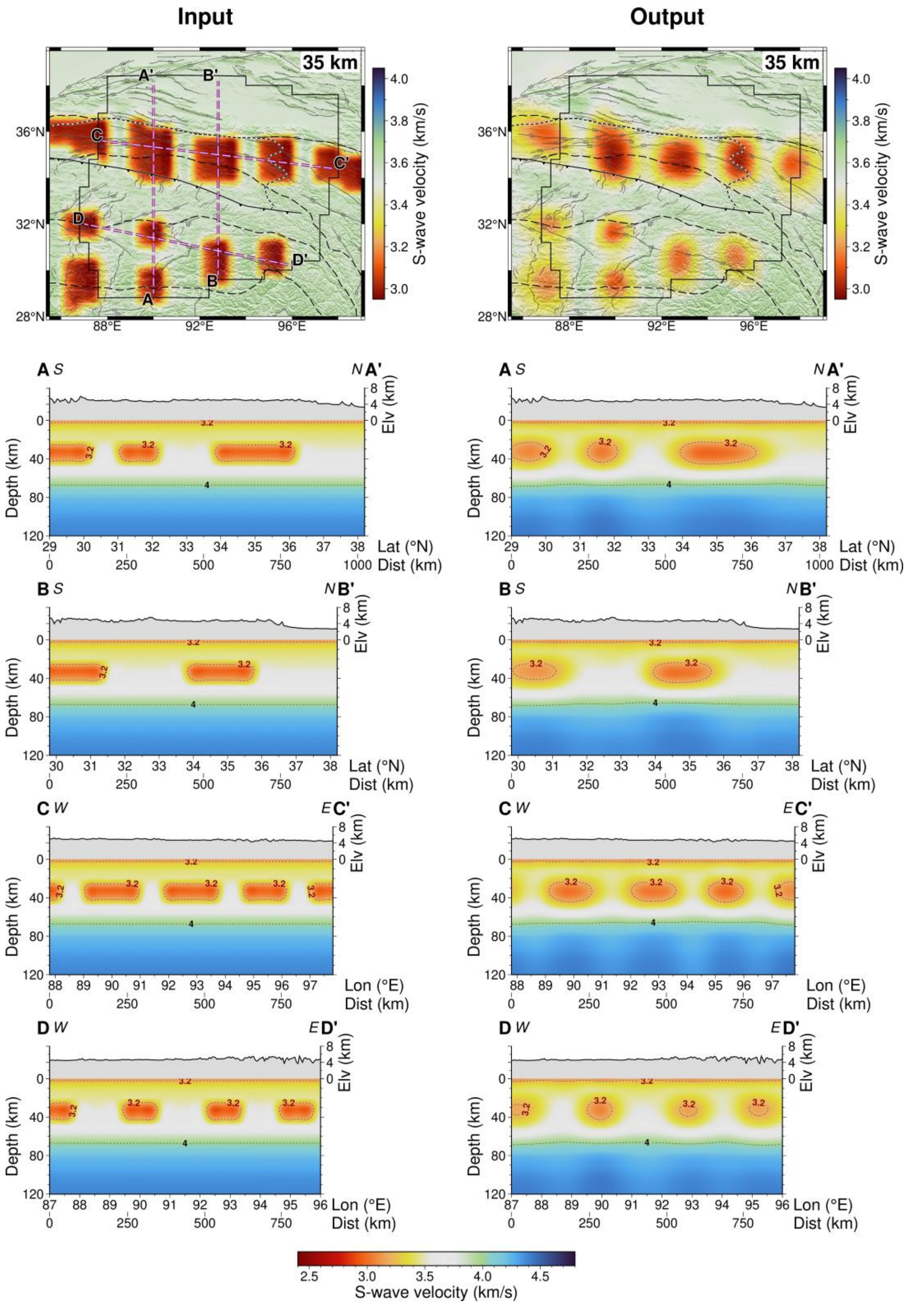

Supplementary Figure 9. Continued for the input model contains segmentally distributed crustal LVZs in north Tibet and six isolated crustal LVZs in south Tibet.

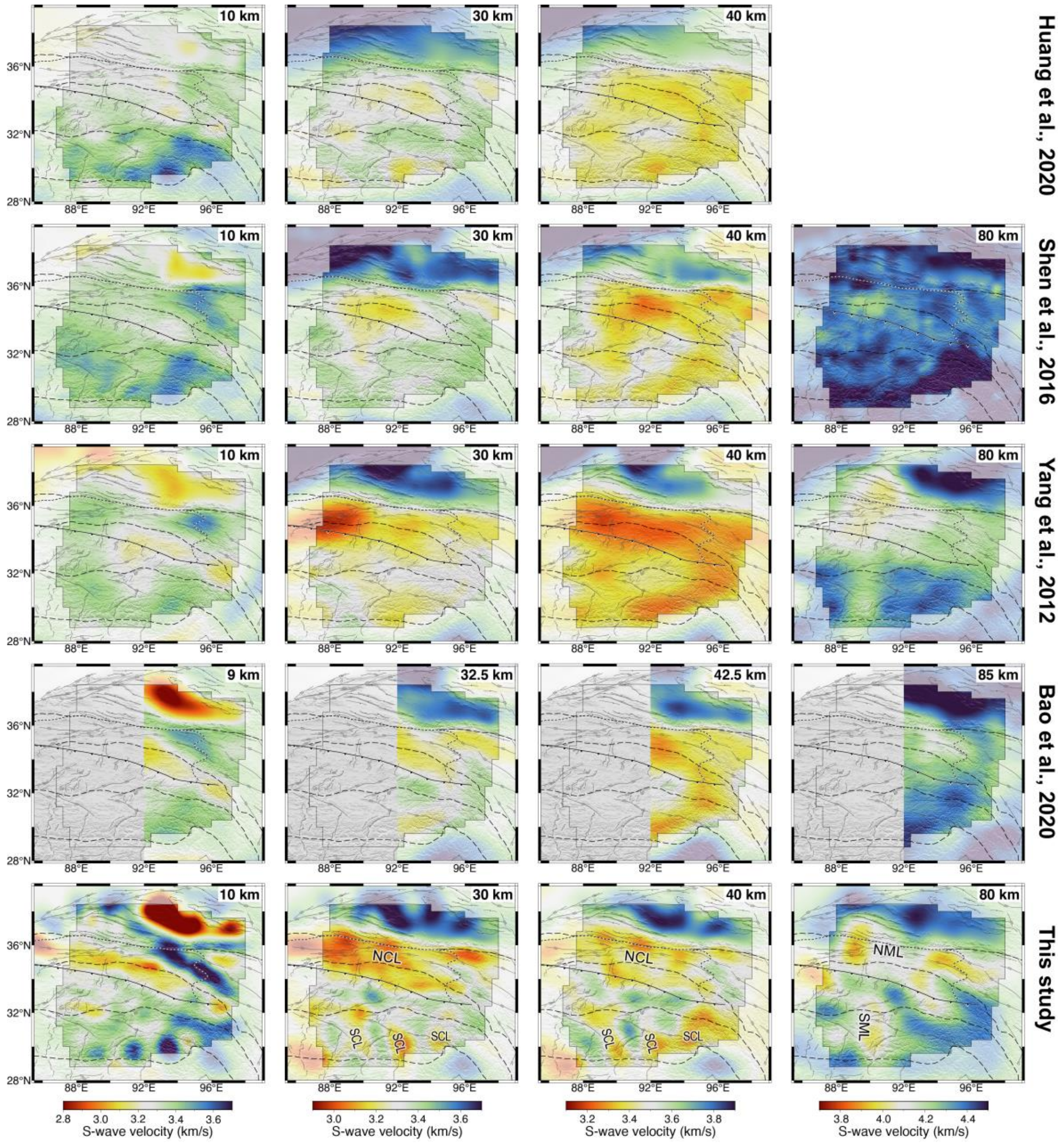

Supplementary Figure 10. Comparing Vs models of the crust and uppermost mantle in the Tibetan Plateau from this study and previous studies, which are from ambient noise tomography of study regions including the whole or the part of Tibetan Plateau [11–14].

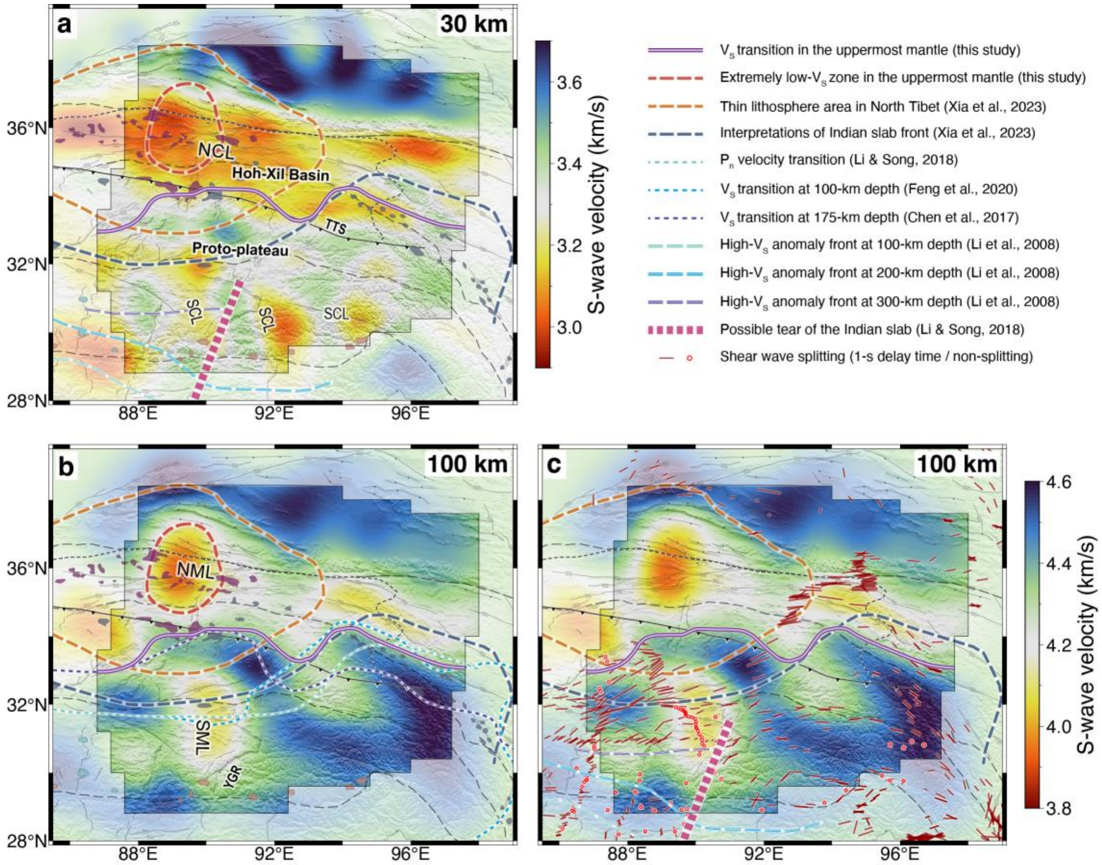

Supplementary Figure 11. Comparison of the crust and upper mantle structures with previous studies. **(a)** The spatial relationship between crustal LVZs (slice at 30 km depth) and mantle structures, including the behavior of the underthrusted Indian slab [15,16] and the north-south structural discrepancy across the Tibetan Plateau. **(b)** The north-south discrepant velocity structure of the uppermost mantle across the Tibetan Plateau constrained by this study (slice at 100 km depth) and previous models from P_n velocity tomography [16], surface wave tomography [17], and full waveform inversion [18]. **(c)** Shear wave splitting measurements from SplitDB [19] compared with the uppermost mantle anomalies (slice at 100 km depth) and the behavior of the underthrusted Indian slab [15,16]. Cenozoic magmatic rocks and tectonics abbreviations are the same as in Fig. 1a.

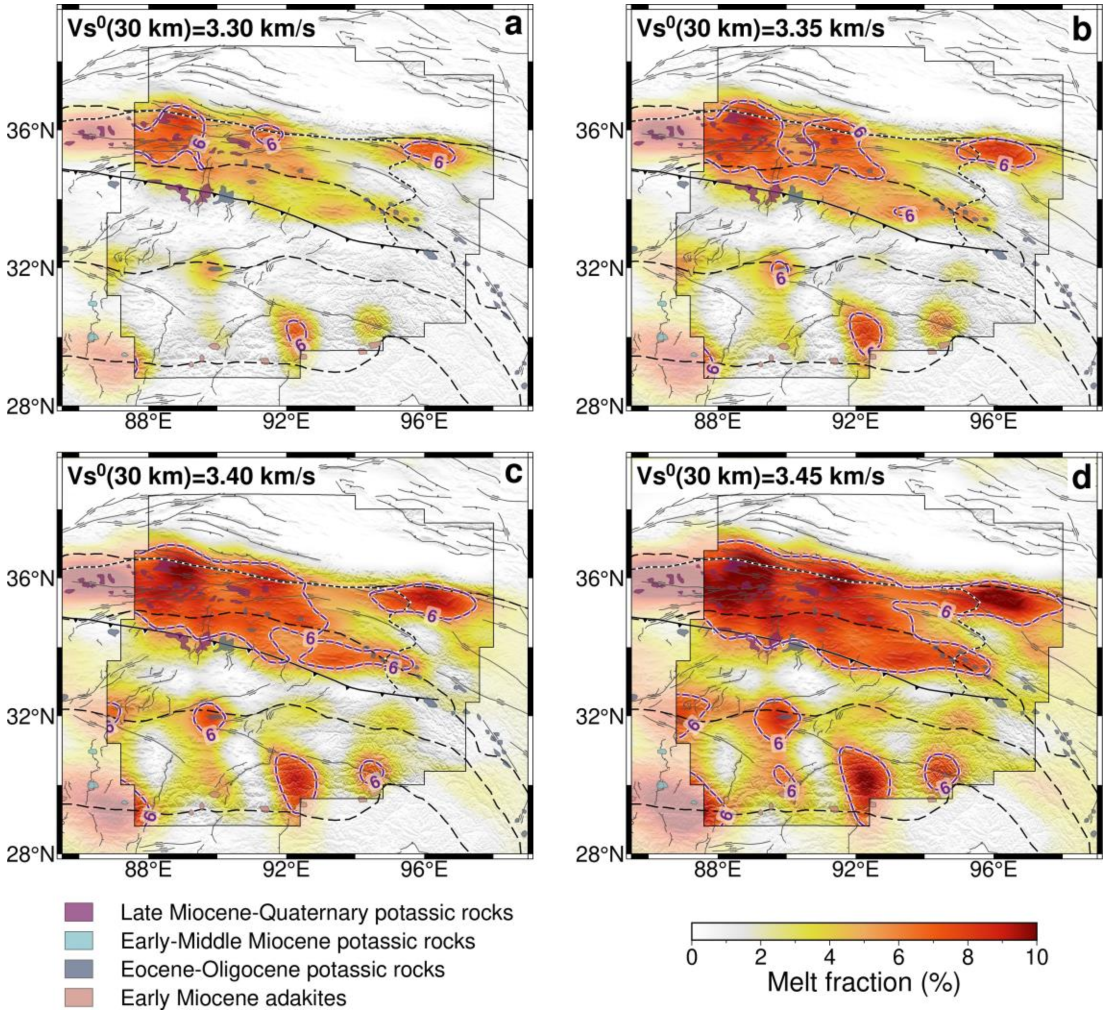

Supplementary Figure 12. Comparison of the melt fractions at 30-km depth calculated with different reference V_S^0 of 3.30 km/s **(a)**, 3.35 km/s **(b)**, 3.40 km/s **(c)**, and 3.45 km/s **(d)**.

Supplementary References

1. Ni J. Deep structure of the Northeastern Tibet collision zone: INDEPTH IV. *Int Fed Digit Seismogr Netw* 2007, DOI: 10.7914/SN/X4_2007.

2. Bensen GD, Ritzwoller MH, Barmin MP *et al.* Processing seismic ambient noise data to obtain reliable broad-band surface wave dispersion measurements. *Geophys J Int* 2007;**169**:1239–60.

3. Ritzwoller MH, Feng L. Overview of pre-and post-processing of ambient noise correlations. In: Nakata N, Gualtieri L, Fichtner A (eds.). *Seismic Ambient Noise*. Cambridge: Cambridge University Press, 2019, 144–87.

4. Fang H, Yao H, Zhang H *et al.* Direct inversion of surface wave dispersion for three-dimensional shallow crustal structure based on ray tracing: methodology and application. *Geophys J Int* 2015;**201**:1251–63.

5. Herrmann RB. Computer Programs in Seismology: An evolving tool for instruction and research. *Seismol Res Lett* 2013;**84**:1081–8.

6. Efron B, Tibshirani R. Bootstrap methods for standard errors, confidence intervals, and other measures of statistical accuracy. *Stat Sci* 1986;**1**:54–75.

7. Takei Y. Effect of pore geometry on V_P/V_S: From equilibrium geometry to crack. *J Geophys Res Solid Earth* 2002;**107**:2043.

8. Yoshino T, Takei Y, Wark DA *et al.* Grain boundary wetness of texturally equilibrated rocks, with implications for seismic properties of the upper mantle. *J Geophys Res Solid Earth* 2005;**110**:B08205.

9. Hacker BR, Ritzwoller MH, Xie J. Partially melted, mica-bearing crust in Central Tibet. *Tectonics* 2014;**33**:1408–24.

10. Kennett BLN, Engdahl ER. Traveltimes for global earthquake location and phase identification. *Geophys J Int* 1991;**105**:429–65.

11. Yang Y, Ritzwoller MH, Zheng Y *et al.* A synoptic view of the distribution and connectivity of the mid-crustal low velocity zone beneath Tibet. *J Geophys Res Solid Earth* 2012;**117**:B04303.

12. Bao X, Song X, Eaton DW *et al.* Episodic lithospheric deformation in Eastern Tibet inferred from seismic anisotropy. *Geophys Res Lett* 2020;**47**, DOI: 10.1029/2019gl085721.

13. Shen W, Ritzwoller MH, Kang D *et al.* A seismic reference model for the crust and uppermost mantle beneath China from surface wave dispersion. *Geophys J Int* 2016;**206**:954–79.

14. Huang S, Yao H, Lu Z *et al.* High-resolution 3-D shear-wave velocity model of the Tibetan Plateau: Implications for crustal deformation and porphyry Cu deposit formation. *J Geophys Res Solid Earth* 2020;**125**:e2019JB019215.

15. Li C, Van der Hilst RD, Meltzer AS *et al.* Subduction of the Indian lithosphere beneath the Tibetan Plateau and Burma. *Earth Planet Sci Lett* 2008;**274**:157–68.

16. Li J, Song X. Tearing of Indian mantle lithosphere from high-resolution seismic images and its implications for lithosphere coupling in southern Tibet. *Proc Natl Acad Sci* 2018;**115**:8296–300.

17. Feng M, An M, Mechie J *et al.* Lithospheric structures of and tectonic implications for the central–east Tibetan plateau inferred from joint tomography of receiver functions and surface waves. *Geophys J Int* 2020;**223**:1688–707.

18. Chen M, Niu F, Tromp J *et al.* Lithospheric foundering and underthrusting imaged beneath Tibet. *Nat Commun* 2017;**8**:15659.

19. Wüstefeld A, Bokelmann G, Barruol G *et al.* Identifying global seismic anisotropy patterns by correlating shear-wave splitting and surface-wave data. *Phys Earth Planet Inter* 2009;**176**:198–212.
